# Changes of Socio-demographic data of clients seeking genetic counseling for hereditary breast and ovarian cancer due to the “Angelina Jolie Effect”

**DOI:** 10.1186/s12885-016-2472-1

**Published:** 2016-07-08

**Authors:** Christine Staudigl, Georg Pfeiler, Katharina Hrauda, Romana Renz, Andreas Berger, Renate Lichtenschopf, Christian F. Singer, Muy-Kheng M. Tea

**Affiliations:** Department of Obstetrics and Gynecology, Division of Senology, Comprehensive Cancer Center, Medical University of Vienna, Waehringer Guertel 18-20, 1090 Vienna, Austria; Department of Obstetrics and Gynecology, Hospital of the Sisters of Charity Linz, 4020 Linz, Austria

**Keywords:** BRCA, Hereditary breast and ovarian cancer, Genetic counseling, Angelina Jolie, Socio-demographic data

## Abstract

**Background:**

The purpose of this study was to evaluate socio-demographic characteristics of clients claiming genetic counseling for hereditary breast and ovarian cancer (HBOC) in Austria. Furthermore, changes of these parameters before and after Angelina Jolie’s (AJ) disclosure of carrying a BRCA mutation were evaluated.

**Methods:**

In this prospective, nonrandomized study 268 consecutive clients seeking genetic counseling for HBOC at the Medical University of Vienna, Department of Obstetrics and Gynecology, Vienna, Austria between June 2012 and June 2014 were included. Socio-demographic data and source of information about HBOC and genetic counseling were evaluated. First, socio-demographic parameters were compared to the general Austrian population. Second, changes in these parameters after AJ’s public disclosure of carrying a BRCA mutation were analyzed.

**Results:**

Subjects were more frequent female, younger and higher educated in comparison to Austria’s general population (*p* < 0.001). Furthermore, level of education in participants was higher before than after AJ’s disclosure (*p* = 0.046). Most clients were informed about genetic counseling by physicians. As expected, after AJ’s public announcement patients were more frequent advised to genetic counseling by social media (*p* = 0.043) and family or friends (*p* = 0.010) than before.

**Conclusions:**

In this present study we could demonstrate that particularly younger and female participants with high educational level attended significantly more often genetic counseling for HBOC. Increased presence of HBOC in media since AJ’s disclosure of carrying a BRCA mutation had lead that information and awareness about HBOC was obtained by a wider audience from different social background.

## Background

In Europe, breast cancer is the most frequently diagnosed cancer in women with 464 000 new cases diagnosed in 2012 [1]. Generally, lifetime-risk of developing breast cancer (BC) is about 12-13 % [2]. BC is mainly a sporadic disease and only 7-15 % of all BC cases are thought to be inherited [3, 4]. About 40-60 % of hereditary breast and ovarian cancers (HBOC) are due to the presence of germline mutations in the breast cancer susceptibility genes type 1 and 2 (BRCA1 and BRCA2) [5]. BRCA mutations are associated with early onset disease and distinct elevated risk of developing BC and ovarian cancer (OC) [6]. The cumulative lifetime risk of BRCA1 mutation carriers is up to 85 % for BC and 20-40 % for OC, whereas BRCA2 mutations carriers have somewhat lesser risk for BC (45-84 %) and a risk up to 31 % for OC [7–11].

Genetic counseling and testing for BRCA1 and BRCA2 mutations is recommended for members of families with familial clustering of BC and/or OC. In Austria, clients have to fulfill specific criteria of medical and / or familial history to enable insurance covered genetic testing. Therefore, individual guidelines exist [12, 13]. Identification of subjects at risk for HBOC is necessary in order to offer distinct strategies to deal with this elevated risk. First, intensified surveillance to allow earlier cancer detection can be offered. Furthermore, risk reducing procedures like bilateral mastectomy and / or salpingo-oophorectomy are obtainable [14, 15].

In the past, celebrities who reported in public media about their personal medical history had an impact on utilization of health service and screening programs [16, 17]. For example Kylie Minogue who reported in public media about her breast cancer led to an increase in bookings of mammographies [18, 19]. On May 14th 2013, Angelina Jolie (AJ) announced in *The New York Time* that she is carrying a BRCA1 mutation and therefore she underwent a prophylactic bilateral mastectomy. The following enormous media attention caused an increased interest and awareness on the topic of HBOC which is called the "Angelina Jolie effect" [20]. This effect led to an increase of referrals for genetic counseling and testing [21–24]. Indeed already in 1998, Mogilner et al. and more recently in 2010, Mac New et al. demonstrated, that the awareness of BRCA1 and BRCA2 and genetic testing has not reached the population uniformly [22, 25]. Especially less well educated people and ethnical subgroups like African Americans were shown to be less informed about HBOC and genetic counseling [22, 26]. Awareness of HBOC and the possibility of genetic counseling and testing can help subgroups which are less well informed to gain awareness about the issue of HBOC.

The aim of this study was to evaluate which population subgroups in Austria are aware about HBOC and therefore attend genetic counseling. Thus, we analyzed socio-demographic data of people who claimed genetic counseling and compared these parameters to the general Austrian population. Furthermore, we investigated if socio-demographic characteristics had changed after AJ's public announcement of carrying a BRCA mutation followed by bilateral prophylactic mastectomy on May 14th 2013.

## Methods

### Participants

In the present single-center study, a total of 268 consecutive women and men who visited the consulting center for HBOC at the Medical University of Vienna, Department of Obstetrics and Gynecology, Vienna, Austria, between June 2012 and June 2014 were included. Socio-demographic data of clients were compared to Austria’s general population. Data about Austria’s population was provided by Statistics Austria [27–33]. Furthermore, we assessed whether socio-demographic characteristics changed after AJ’s announcement of carrying a BRCA mutation due to the “Angelina Jolie Effect”.

The study was performed in accordance with the regulations of the declaration of Helsinki and was approved by the institutional review board of the Ethics Committee of the Medical University of Vienna (IRB approval number: 1292/2012).

Only participants providing specific written informed consent to participate in the study after physician elucidation were included in this study and invited to complete the socio-demographic questionnaire. Afterwards subjects received standardized genetic counseling. Risk assessment for potential BRCA mutation was evaluated using family history and if possible a three to four-generation pedigree.

### Questionnaire

We designed the questionnaire about the socio-demographic data as follows: information about age at time of study enrollment, gender (male vs. female), nationality (free-text), first language (free-text) and religious confession (subdivided into Roman-Catholic, Evangelic, creedless and other confessions) was asked. Furthermore, data about marital status (dichotomized in married/cohabitating or single including divorced and widowed), number of people living in the household (free-text) and number of children (free-text) was asked. Moreover, information about educational attainment was obtained. Concerning this question the questionnaire was subdivided in two categories: basic-educated including compulsory school, finished apprenticeship-training, finished intermediate technical or vocational school, secondary school, post-secondary college or college and high-educated defined as holding an university degree. Besides employment status (dichotomized in employed or unemployed) net monthly income (subdivided in ≤1000 Euro (€), 1001-2000€ and ≥2001€) was asked.

Additionally, we assessed the source how participants learned about HBOC and genetic counseling (subdivided in referral from a physician like a gynecologist, radiologist, general practitioner or another healthcare specialist or public media like television, internet, radio and social environment like family and friends). In this category multiple answers were possible.

### Statistical analysis

The analysis was carried out by using descriptive statistics. Variables are described by mean (standard deviation, SD) when normally distributed. Pearson’s Chi-Square test with Bonferroni correction or students T- test according to the scale of variable (categorical or continuous) was used to assess differences between the expected- and the observed frequencies of collected parameters. Results are based on two-sided tests and *p*-values of <0.05 were considered statistically significant. Statistical analysis was performed using IBM SPSS (Statistical Package for Social Sciences) for Windows, Version 23.

## Results

A total of 268 clients were included in the study and completed the socio-demographic questionnaire during 2012–2014. Out of all participants 158 (59.0 %) were included before AJ’s disclosure of carrying a BRCA mutation in May 2013 and 110 (41.0 %) afterwards. For the following statistical analysis all missing data were excluded.

Subject’s mean age at the time of enrollment was 39.8 years (SD 12.6). As expected, the majority of participants were females 264 (98.5 %), whereas only four clients (1.5 %) were males. Three male subjects participated before AJ’s disclosure and one afterwards.

As expected, the majority of 233 (87.9 %) participants were Austrian citizens, whereas 12 (4.5 %) were citizens of Germany and each 3 (1.1 %) were Serbians or Turkish. Each two (0.8 %) participants were from Croatia, Poland, Romania or Hungary, respectively. Only six (2.3 %) participants were from not specified countries.

The most common first-language of study-participants was German (*n* = 233, 86.9 %). Other represented languages varied strongly with five (1.9 %) participants indicated Serbian and each four (1.5 %) participants quoted Croatian, Hungarian, Polish or Turkish as their mother language. Other 14 (5.2 %) spoke other previously not mentioned native languages.

Regarding the religious confession 145 (54.1 %) were Roman Catholic, 23 (8.6 %) were Protestants, 75 (28.0 %) were creedless and 22 (8.3 %) belonged to other religious communities.

Regarding the marital status, the majority 179 (66.8 %) of participants was married or in a partnership whereas one third 89 (33.2 %) was single, divorced or widowed.

The mean number of people in this study living in the household was 2.66 (SD 1.4, minimum (min.) 1, maximum (max.) 10). Mean number of biological children was 1.04 (1.1 SD, min. 0 max. 4), respectively. In detail 112 (43.4 %) clients were childless whereas 119 (46.1 %) stated to have one or two children and only 27 (10.4 %) had three or more children at time of study survey.

**Fig. 1:**
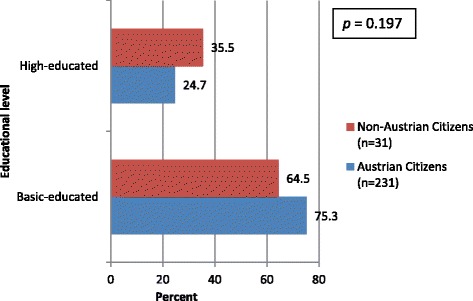
Educational-level in dependence of citizenship (*n* = 268)

Within our study-population 195 (73.6 %) were basic-educated whereas 70 (26.4 %) were high-educated with a university degree. Differences of educational-level in dependence of citizenship are presented in Fig. 1.

Overall, 237 (90.8 %) subjects were currently employed, whereas 24 (9.2 %) were unemployed at the time of study survey. In detail 86 (33.9 %) participants earned less than 1000 € per month in their job. The majority of 117 (46.1 %) had an average monthly income from 1001 – 2000 € and 51 (20.1 %) participants earned more than 2000€ per month.

Differences of study participants compared to the general Austrian population are demonstrated in Table 1.

**Table 1:** Socio-demographic data of study population compared to general Austrian population

Parameter	Study population (*n* = 268)	General Austrian population [27–33]	*p*-Value^+^
Age mean in years (SD)	39.8 (SD 12.6)	49.46 (NA)	< *0.001*
Gender n (%)			< *0.001*
Female	264 (98.4 %)	4 367 382 (51.1 %)	
Male	4 (1.6 %)	4 176 550 (48.9 %)	
Citizenship n (%)			*0.655*
Austria	233 (87.9 %)	7 440 084 (87.0 %)	
Other than Austrian	32 (12.1 %)	1 103 848 (13.0 %)	
First Language n (%)			*0.204*
German	233 (86.9 %)	3940.1 (84.1 %)	
Other than German	35 (13.1 %)	745.2 (15.9 %)	
Religious confession n (%)			< *0.001*
Roman-Catholic	145 (54.1 %)	5 917 274 (73.6 %)	
Evangelic	23 (8.6 %)	376 150 (4.7 %)	
Other confessions	22 (8.2 %)	615 577 (7.7 %)	
Creedless	75 (28.0 %)	1 123 925 (14.0 %)	
Number of Children mean (SD)	1.02 (SD)	1.46 (NA)	< *0.001*
Educational level n (%)			< *0.001*
Basic-educated	195 (73.6 %)	4 103 107 (87.5 %)	
High-educated	70 (26.4 %)	584 448 (12.5 %)	
Employment status n (%)			
Unemployed	12 (4.8)	NA	
Employed	237 (95.2)	NA	
Net monthly income n (%)			
0 – 1000€	86 (33.9)	NA	
1001 – 2000€	117 (46.0)	NA	
2001 – > 3000€	51 (20.1)	NA	

*n* number, *NA* not applicable, *SD* standard deviation

^+^Pearson’s Chi-Square test, *p* is considered significant when < 0.05

Analysis of socio-demographic data before and after AJ’s disclosure of carrying a BRCA mutation showed that portion of high-educated clients significantly decreased after her disclosure (p = 0.046, Pearson’s Chi-Square test). No difference in subjects’ mean age between study-groups who participated before AJ’s disclosure (mean age 39.34 years, SD12.7) and afterwards (mean age 40.84 years, SD 12.8) was found (*p* = 0.291, Pearson’s Chi-Square test). Details about changes in socio-demographic characteristics before and after AJ’s public announcement are given in Table 2.

**Table 2:** Socio-demographic data before and after Angelina Jolie’s (AJ) public announcement of carrying a BRCA mutation via social media

Parameter	Before AJ’s disclosure *n* = 158	After AJ’s disclosure *n* = 110	*p*-Value^+^
Age mean (SD)	39.34 (12.7)	40.84 (12.8)	*0.291*
Citizenship n (%)			*0.299*
Austria	139 (89.7 %)	94 (85.5 %)	
Other than Austria	16 (10.3 %)	16 (14.5 %)	
First Language n (%)			*0.893*
German	137 (86.7 %)	96 (87.0 %)	
Other than German	21 (13.3 %)	14 (12.7 %)	
Religious confession n (%)			*0.296*
Roman-Catholic	84 (53.2 %)	61 (57.0 %)	
Evangelic	15 (9.5 %)	8 (7.5 %)	
Creedless	46 (29.1 %)	29 (27.1 %)	
Other confessions	13 (8.2 %)	9 (8.4 %)	
Marital Status n (%)			*0.239*
Married or Cohabitating	110 (69.6 %)	69 (62.7 %)	
Single/Divorced/Widowed	48 (30.4 %)	41 (37.3 %)	
Number of Children mean (SD)	1.0 (1.1)	1.1 (1.1)	*0.540*
Educational level n (%)			*0.046*
Basic-educated	107 (69.0 %)	88 (80.0 %)	
High-educated	48 (31.0 %)	22 (20.0 %)	
Employment status n (%)			*0.169*
Unemployed	11 (7.1 %)	1 (12.1 %)	
Employed	143 (92.9 %)	94 (87.9 %)	
Net monthly income n (%)			*0.735*
0 – 1000€	49 (32.0 %)	37 (36.6 %)	
1001 – 2000€	73 (47.7 %)	44 (43.6 %)	
2001 – >3000€	31 (20.3 %)	20 (19.8 %)	

^+^Pearson’s Chi-Square test, *n* = number, *SD* = standard deviation

**Fig. 2:**
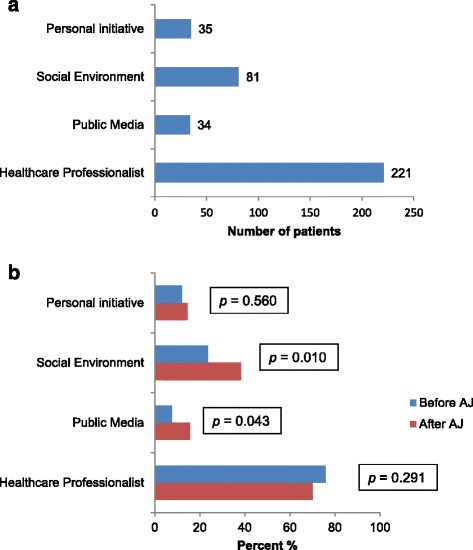
Source of Information about genetic counseling for hereditary breast and ovarian cancer of all patients (**a**), differences before and after the announcement of Angelina Jolie carrying a BRCA mutation (**b**)

Regarding the question of source of information about HBOC multiple answers were possible. Altogether 221 (82.8 %) participants were referred to genetic counseling by their physicians, 142 (64.3 %) by gynecologists, 27 (12.2 %) by radiologists, 15 (6.8 %) heard about genetic counseling from their primary health care provider and 37 (16.7 %) got a referral from another healthcare specialist. Furthermore, 34 (12.7 %) subjects mentioned social media of which 11 (32.4 %) were television, 3 (8.8 %) radio and 20 (58.8 %) internet, whereas 81 (30.3 %) obtained family members and friends as source of information. Moreover 35 (13.3 %) participants stated personal initiative as source of information for genetic counseling. Data about differences how participants get to know about genetic counseling and differences before and after AJ’s public announcement of carrying a BRCA mutation via social media are given in Fig. 2.

## Discussion

Genetic counseling and testing for HBOC is recommended for people with familial clustering of BC and / or OC. In the present study we investigated which population groups are already informed about HBOC and attended genetic counseling at our institute. We were able to demonstrate that compared to the general Austrian population significantly more often female, younger and higher educated as well as creedless clients attend genetic counseling.

Since AJ published in social media that she is carrying a BRCA mutation and therefore had a bilateral prophylactic mastectomy, interest on genetic counseling and HBOC increased enormously at our institution. This finding is consistent with already published data showing that AJ’s disclosure led to an increased global interest and awareness on HBOC, BRCA gene mutations and genetic counseling for HBOC the so-called “Angelina Jolie Effect” [20, 21, 23]. Similar “Celebrity” effects have already been described after other famous personalities reported in public media about their personal medical history. In 2005 Kylie Minogue reported about her breast cancer disease which led to an increase of mammography referrals in Australia [18]. Furthermore, after Jade Goody reported about her cervical cancer diagnosis, cervical cancer screening attendance increased significantly [16].

Already in 2001, Lodder et al. reported, that men were less likely to opt for genetic counseling for HBOC compared to women [34]. Although this study is more than 10 years old, this result is still congruent with our findings. In total, only four men sought genetic counseling in our department within the two years of study period. Furthermore, in our study the rate of male participants did not increased after public reporting about AJ’s BRCA mutation. Consequently, reporting in social media had hardly any effect on the number of genetic consultations of men in our study population. Because of the small sample size in our study general conclusions cannot be made. Lifetime risk for male mutation carriers is up to 6 % for breast cancer (BRCA 2) and only slightly increased for other types of cancer. However, due to autosomal dominant inheritance of BRCA genes, every descendant has a 50 % risk of inheriting a BRCA mutation [35, 36]. Therefore in high-risk families, genetic counseling and testing is recommended also for men. These facts show the importance that also men from families with clustering of BC and / or OC obtain information by a well-informed physician as well as become objectives of information- events in order to call attention and opt for genetic testing.

In 2004 and 2005 two studies showed that awareness about HBOC and genetic testing are differing by race [37, 38]. In contrast to this finding, in our study population no differences in distribution of nationalities compared to the general Austrian population could be shown (*p* = 0.655, Pearson’s Chi Square Test).

A significant difference of religious confession compared to the general Austrian population (*p* = 0.001, Pearson’s Chi-Square test) was shown in our study. Possibly, there is an association between the participants’ belonging to a religious community or being creedless influencing the subjects’ perception and attitude about genetic testing [39]. Due to the lack of ethnical and social background of the study population, the change in statements about community of religion after increased reports about HBOC in social media remains questionable for further surveys.

Mogilner et al. and MacNew et al. reported that higher educated people and people with higher income have greater knowledge about breast cancer genes and genetic testing compared to lower educated people or people with lower income [25, 40]. Additionally, health literacy and health numeracy may be essential in understanding the opportunity of genetic counseling and cancer prevention programs [41]. The high educational background of our study participants compared to general population suggests the assumption that education may provide people with the knowledge, skills and confidence to look for specific information and as a consequence attend genetic counseling. Moreover, due to the “Angelina Jolie Effect” more clients with lower educational level were interested in genetic counseling through social media and personal environment (family and friends) than before. The increasing number of lower educated participants after AJ’s disclosure may show the impact of celebrities like AJ and social media on the awareness about the issue of HBOC in the general population and less well educated people. A comparable effect was already seen in 1987 after Nancy Reagan’s mastectomy, thereafter it was a temporary effect that women were less likely to opt for breast conserving surgery than before. The influence of her disclosure on health-care decisions was the strongest among women with lower educational status and income [42]. It seems that celebrities reporting about health topics in social media reach a large audience.

Recent studies showed that after AJ's disclosure the general knowledge and understanding of HBOC did not increase but the number of people asking for genetic counseling who are not characterized as at elevated risk and therefore do not need genetic counseling and risk-reducing procedures increased [23]. Generally, it is relevant to know which social subgroups are at an increased risk for developing BC and/or OC attend genetic counseling and for which social subgroups more intensive interventions are necessary to enable sufficient awareness and information about the issue of HBOC and genetic counseling.

Regarding the source of information about HBOC, two studies showed that participants of their surveys obtained information mainly from television and radio [25, 40]. More recently, Juthe et al. showed that AJ’s announcement strongly increased the search of information in online resources like twitter and the homepage of the National Cancer Institute [24]. In agreement with these findings our results showed that social media like internet and television were mentioned significantly more often after AJ’s announcement than before (p = 0.043, Pearson’s Chi-Square test). Furthermore, social environment especially family members were more often involved in information processing. In this context families are often talking to each other about the topic of HBOC but also may be hesitant to due to possible implications to other family members. AJ encouraging family involvement is another positive message we are seeing in the media and we know from recent research that family openness about this topic is critical to family functioning as well as prevention [43, 44].

Although in our study the rate of information from social media increased after AJ’s disclosure the main source of information are still physicians. Data about U.S. adults from 2002–2008 provided by the Health Information National Trends Survey show that respondents use the Internet first for specific cancer information. Additionally this tendency increased during study period. Interestingly, over the same period of time trust in health information provided from the internet decreased while trust in information from physicians increased [45].

Although this study has substantial strengths, like analyzing prospective socio-demographic data of patients attending genetic counseling for HBOC, it also has limitations. The study was performed as a single center study at the University Hospital in Vienna, Austria, so mainly inhabitants living in and around Vienna are represented in this analysis. In addition to that, due to the fact that the genetic counseling center is part of the Department for Gynecology and Obstetrics men seeking for genetic counseling might be underrepresented in our study population. Furthermore, the appointments for genetic counseling in our department are scheduled about 6 weeks in advance, so effects on data after AJ’s disclosure might be greater than demonstrated because appointments were already scheduled. Unfortunately, a comparison of data on employment status and income of Austria’s general population and the study population was not possible. Furthermore, we were not able to provide the information in terms of personal and familial history specifically for this study since it was not the goal of the study to distinguish between low and high risk individuals. It was not part of the ethical consent to use this data.

## Conclusion

In conclusion in this present study we could demonstrate that younger and particularly female participants with higher educational level attended significantly more often genetic counseling for HBOC. The presence of HBOC in media in general and in Austria since AJ’s disclosure of carrying a BRCA mutation and her bilateral prophylactic mastectomy led that more awareness about HBOC was obtained by a wider audience (The Angelina Jolie Effect). In addition to the conventional information through physicians, we conclude that also information in media like internet and television is becoming more important to provide information to as many people as possible from different social background.

## Abbreviations

AJ, Angelina Jolie; BC, breast cancer; BRCA 1, BRCA 2, germline mutations in the breast cancer susceptibility genes type 1 and 2; HBOC, hereditary breast and ovarian cancer; n, number; NA, not applicable; OC, ovarian cancer; SD, standard deviation; U.S., United States of America
